# A Differentiation-Related Gene Prognostic Index Contributes to Prognosis and Immunotherapy Evaluation in Patients with Hepatocellular Carcinoma

**DOI:** 10.3390/cells11152302

**Published:** 2022-07-26

**Authors:** Jingjing Xiao, Tao Liu, Zhenhua Liu, Chuan Xiao, Jun Du, Shi Zuo, Haiyang Li, Huajian Gu

**Affiliations:** 1 School of Clinical Medicine, Guizhou Medical University, Guiyang 550000, China; xiaojingjing@gz5055.com (J.X.); tiger1986@stu.gmc.edu.cn (T.L.); xiaochuan@stu.gmc.edu.cn (C.X.); dujun@gzykdxfsyyxew.wecom.work (J.D.); drzuoshi@gmc.edu.cn (S.Z.); lihaiyang@gmc.edu.cn (H.L.); 2 Department of Hepatobiliary Surgery, Guizhou Provincial People’s Hospital, Guiyang 550002, China; liuzhenhua@gz5055.com; 3 Department of Pediatric Surgery, The Affiliated Hospital of Guizhou Medical University, Guiyang 550000, China; 4 Department of Hepatobiliary Surgery, The Central Hospital of Enshi Tujia and Miao Autonomous Prefecture, Enshi 445000, China; 5 Department of Hepatobiliary Surgery, Affiliated Hospital of Guizhou Medical University, Guiyang 550000, China

**Keywords:** hepatocellular carcinoma, differentiation trajectory, differentiation-related gene prognostic index, immune checkpoint inhibitor, immune infiltration

## Abstract

Hepatocellular carcinoma (HCC) is the most common gastrointestinal tumor with a poor prognosis, which is associated with poor differentiation of tumor cells. However, the potential value of cell differentiation-related molecules in predicting the benefit and prognosis of immune checkpoint inhibitors (ICI) therapy remains unknown. Herein, to investigate the differentiation trajectory of HCC cells and their clinical significance, a differentiation-related gene prognostic index (DRGPI) based on HCC differentiation-related genes (HDRGs) was constructed to elucidate the immune characteristics and therapeutic benefits of ICI in the HCC subgroup defined by DRGPI. Single-cell RNA sequencing (scRNA-seq) and bulk RNA-seq data from four HCC samples were integrated for bioinformatics analysis. Then, PON1, ADH4, SQSTM1, HSP90AA1, and STMN1 were screened out to construct a DRGPI. More intriguingly, RT-qPCR validation of the expression of these genes yielded consistent results with the TCGA database. Next, the risk scoring (RS) constructed based on DRGPI suggested that the overall survival (OS) of the DRGPI-high patients was significantly worse than that of the DRGPI-low patients. A nomogram was constructed based on DRGPI-RS and clinical characteristics, which showed strong predictive performance and high accuracy. The comprehensive results indicated that a low DRGPI score was associated with low TP53 mutation rates, high CD8 T cell infiltration, and more benefit from ICI therapy. Homoplastically, the high DRGPI score reflected the opposite results. Taken together, our study highlights the significance of HCC cell differentiation in predicting prognosis, indicating immune characteristics, and understanding the therapeutic benefits of ICI, and suggests that DRGPI is a valuable prognostic biomarker for HCC.

## 1. Introduction

Hepatocellular carcinoma (HCC) is one of the most common malignancies worldwide and a leading cause of cancer-related death [1]. Hepatectomy is the most promising treatment for early HCC [2]. However, the 5-year recurrence rate after hepatectomy is as high as 50–70%, and the 5-year survival rate is only 60%, indicating a poor prognosis [3]. Recently, molecularly targeted drugs and immunotherapy represented by immune checkpoint inhibitor (ICI) therapy have shown encouraging changes in the treatment of HCC [4,5]. ICIs have been shown to prolong overall survival (OS) to some extent in advanced HCC patients who are sorafenib-intolerant or treatment-naïve [6]. Some studies have shown that the objective remission rate (ORR) of ICIs for hepatocellular carcinoma is about 17–20% [7], and their ORR is low, so it is vital to discover predictive markers for the benefit of ICI therapy [8]. However, due to the complexity and diversity of the tumor immune microenvironment, immunotherapy or combined immunotherapy has not fundamentally changed the therapeutic outcome of patients with HCC [9], and there is a lack of biomarkers to predict the outcome of ICI therapy [10,11]. Therefore, it is extremely urgent to understand the composition and characteristics of the HCC immune microenvironment and to find biomarkers that can predict the benefit of ICI therapy in patients with HCC, which will also provide a favorable basis for individualized ICI therapy for patients with HCC.

As an emerging technology, single-cell RNA sequencing (scRNA-seq) has extensively revealed the complex functions and mechanisms of cells [12]. In particular, the differentially expressed genes among individual cells by analyzing the cell differentiation trajectory, which provides a new insight for finding the correlation between the heterogeneity of the immune microenvironment and ICI therapy benefits [13]. Here, based on the analysis of cell differentiation trajectory in scRNA-seq, we identified subtypes of HCC cells in the immune micro-environment and searched for prognostic genes associated with cell differentiation. Meanwhile, weighted gene co-expression network analysis (WGCNA) was used to screen hub genes associated with cell differentiation in HCC samples from The Cancer Genome Atlas (TCGA) database, and a differentiation-related gene prognostic index (DRGPI) and nomogram were constructed to predict the immunotherapy response in patients with HCC. Additionally, the ability of DRGPI to predict prognosis in patient ICI therapy was investigated and compared with other biomarkers, tumor immune dysfunction and rejection (TIDE), and tumor inflammatory features (TIS). Collectively, we developed a useful biomarker and nomogram to accurately predict the prognosis and the benefits of ICI therapy in patients with HCC, suggesting that DRGPI may be a promising prognostic biomarker.

## 2. Materials and Methods

### 2.1. HCC Tissue Specimen Collection

Ten specimens of HCC tissues were surgically removed from the Department of Hepatobiliary Surgery of the Affiliated Hospital of Guizhou Medical University from June 2021 to April 2022, and 10 specimens of normal liver tissues adjacent to cancer that were more than 2 cm away from the HCC tissues were collected. All patients were pathologically confirmed as HCC (Barcelona Clinic Liver Cancer stage A), signed an informed consent form, and approved by the ethics committee.

### 2.2. ScRNA-Seq and Bulk RNA-Seq Data Acquisition and Preprocessing

ScRNA-seq data with a reading depth of 10× genomics (including mRNA expression profiles) of 3200 cells from four HCC samples were obtained from the GSE146115 dataset in the Gene Expression Omnibus (GEO, https://www.ncbi.nlm.nih.gov/geo/) (accessed on 15 July 2021) database and were performed to identify differentiation-related genes. Then, the “Seurat” package of R (v3.5.243) was used to process the scRNA-seq data, and the Percentage Feature Set function was used to filter the cells with ≥5% of mitochondria-expressed genes. Next, cells with a gene number <100 and a sequencing number <50 were filtered using correlation analysis, which was conducted to elucidate the relationship between sequencing depth per cell and total intracellular RNA sequences.

After filtering low-quality cells, the scRNA-seq gene expression dates were normalized by the LogNormalize function, and 1500 feature genes with high cell-to-cell variation were identified using the Find Variable Features method. In addition, gene expression profile information and corresponding clinical information of RNA-seq data from 50 paracancerous tissues and 374 HCC samples were extracted from a publicly available genetic database of cancer patients, TCGA (https://tcga-date.nci.nih.gov/tcga) (accessed on 18 July 2021), as a training dataset. Simultaneously, 260 HCC samples were obtained from the ICGC (https://dcc.icgc.org/donor.LIRI-JP.tsv.gz) (accessed on 20 July 2021) dataset as a validation dataset, among which 28 patients did not have detailed clinical data (e.g., survival time = 0 or unknown; absence of pathological diagnosis) was removed from the study.

### 2.3. Principal Component Analysis and Cell Annotation

To tackle high-dimensional HCC scRNA-seq data, principal component analysis (PCA) was used for processing the high-dimensional scRNA-seq data successfully, which was executed to identify substantially dimensions with a *p*-value < 0.05 and further adjusted by False Discovery Rate (FDR < 0.05). After that, the t-Distributed Stochastic Neighbor Embedding (t-SNE) algorithm was applied for dimensionality reduction by analyzing the top 15 principal components (PCs) and acquiring the major clusters analysis across all cells. Marker genes in each cluster were considered significantly differentially expressed if the log2 fold change (FC) absolute value was ≥1 or <0.5 and the *p*-value was ≤0.05, and visualized the top 10% of marker genes from clusters via heatmaps and two marker genes with violin plot. Then, the “survival” package of R (version 4.0.2) was used to determine and annotate different cell clusters.

### 2.4. HCC Cells Trajectory Analysis and Differentiation-Related Gene Identification

To explore the direction of cell differentiation, the monocle2 algorithm [14] was utilized to analyze pseudo-time trajectories of the HCC cells. Then, the differentially expressed genes in cells with distinct branches were identified using the “DEseq2” package of R (version 4.0.2), and with |log2 (FC)| > 1 and FDR < 0.05 were identified as differentiation-related genes.

### 2.5. Differentiation-Related Gene Classification Based on Patients with HCC in ICGC Cohorts

Transcriptome data of differentiation-related gene expression from the ICGC database were log2-transformed for normalization, and 904 differentiation-related genes were maintained in HCC molecular subtyping. To identify differentiation-related genes subtype-specific of patients with HCC, K-means algorithm consensus clustering and the R package Consensus Cluster Plus were performed, which provide quantitative and visual evidence of stability for estimating the number of unsupervised clusters subtypes. Parameter settings were as follows: max-K = 9, reps = 500, p-Item = 0.8, p-Feature = 1. The best number of clusters was determined using the cumulative density function (CDF) curves of the consensus score and consensus heatmap and by selecting the optimal value of K. Then, weighted gene coexpression network analysis (WGCNA) was used to build co-expression modules of differentiation-related genes and pathological grades module eigengenes and performed using the R package WGCNA. The “survival” package of R was used to determine the Kaplan–Meier survival analysis of different subtypes, and the “ggplot2” package of R was performed to analyze the relationship of the clinicopathological feature between different molecular subtypes. Furthermore, the differences in DRGPIs expressions in different cell differentiation trajectories were visualized using boxplots.

### 2.6. CIBERSORT Estimation and the Expression of Immune Checkpoint Gene (ICGs) in Molecular Subtypes

The stromal score, estimate score, and immune score of HCC samples were computed using the ESTIMATE algorithm. The “CIBERSORT” software calculated the abundance of 22 immune cell subgroups in each sample from the ICGC database. Subsequently, the “limma” package of R was employed for identifying the immune infiltration data of immune cells, including different subtypes of HCC, and the histogram represented the significant differences of immune cells in the HCC samples calculated using the histogram function. Additionally, through an extensive literature survey [15,16], 38 validated ICGs were identified, and the expression of ICGs in the ICGC dataset was identified by differential expression analysis. Then, the prognostic value of immune checkpoint molecules and immune cells was performed by Kaplan–Meier analysis (using log-rank test).

### 2.7. Construction and Validation of a DRGPI and Nomogram

We identified the key modules related to HCC differentiation using the “WGCNA” package of R and selected them as intersecting modules with the highest correlation with tumor grade. Subsequently, the relationship between the expression of DRGPI and the survival of patients with HCC in the TCGA validation cohort was assessed by univariate Cox regression analysis (the significance threshold was *p* < 0.001), and key prognostic-related genes were picked by LASSO and Cox multivariate regression analysis. Then, DRGPIs-based prognostic risk score was calculated as follows: Risk score = expression of Gene1 *β1 + expression of Gene2 *β2 +… expression of GeneN * βn (β, regression coefficient) [17].

Therefore, key prognostic-related DRGPIs were included to construct a risk score model and predict the survival of patients with HCC. Then, patients with HCC in the TCGA database were divided into low-risk groups and high-risk groups. Next, Kaplan–Meier survival analysis was used to calculate the OS of the low-risk and high-risk groups, and the significance threshold was 0.05 by a two-tails log-rank test. Clinical factors (e.g., age, sex, tumor stages, and tumor grade) and RS were used to construct a nomogram by using the “rms” package of R. Additionally, the Harrell’s concordance index (C-index) of the “survival ROC” and “survcomp” of R and receiver operating characteristic (ROC) curve of the “time ROC” package of R was used to compare the prognostic capabilities of the nomogram. Ultimately, the ICGC validation cohort was used to further verify the accuracy of predictive models.

### 2.8. mRNA Extraction and RT-qPCR Analysis

Total RNA was extracted from the tissues with TRIzol reagent (Invitrogen, Carlsbad, CA, USA), reverse transcribed to cDNA, and spiked with SYBR Premix EX Taq II Kit (T akara, Kusatsu, Shiga, Japan), protected from light. The cDNA was synthesized according to the instructions of the polymerase chain reaction (PCR) kit, and the expression of the model gene was amplified using the cDNA as a template. The PCR reaction conditions and reaction system were performed according to the kit’s instructions. The expression level of model genes was normalized to the internal control GAPDH and calculated according to the 2^(−ΔΔCt)^ method. All primer sequences are shown in Supplementary Table S1.

### 2.9. Gene Set Enrichment Analysis (GSEA), Correlation Analysis, and Somatic Mutation Analysis of the DRGPI Subgroups

GSEA (http://software.broadinstitute.org/gsea/index.jsp) (accessed on 2 September 2021) [18] was used to discover functional differences between the low-risk and high-risk groups, and the false discovery rate (FDR) adjusted *p*-value < 0.05 was defined as statistically significant. Additionally, a Single sample GSEA (ssGSEA) analysis was performed using the “GSVA” package of R to obtain several representative gene set scores. To analyze differences in the number and quality of genetic mutations between the low-risk and high-risk groups, dates of genetic alterations were obtained from the cBioPortal database and determined using the “Maftools” package of R. Meanwhile, Pearson’s correlation analysis was applied to determine the relationship between key prognostic genes. Additionally, Spearman rank correlation analysis was used to evaluate the correlation between DRGPI score, PD-L1 expression, and total mutation burden (TMB). Furthermore, difference analysis was used to determine whether different molecular subgroups in ICGC and TCGA databases differ under DRGPI subgroups.

### 2.10. Comprehensive Analysis of Molecular and Immune Characteristics and ICI Therapy of Different DRGPI Subgroups

CIBERSORT algorithm was performed to estimate the relative proportion of 22 infiltrated immune cells in 360 HCC samples, gene signatures were screened to define the immune function between DRGPI subgroups, and ssGSEA was performed to score the gene sets and compare the scores [19]. Then, survival analysis was used to investigate the prognostic value of DRGPI-based subgroups of patients after immunotherapy and to analyze the relationship between the TCGA and ICGC cohorts of anti-PD-L1 therapy [20]. Furthermore, the area under the curve (AUC) of each time-dependent ROC curve of the “timeROC” package of R was used to compare the prognostic values between DRGPI, TIDE, and TIS. Among them, TIS was calculated by log2-transformation of 18 characteristic genes using normalized expression mean [21], and the TIDE score was analyzed using online software (http://tide.dfci.harvard.edu/) (accessed on 5 September 2021).

### 2.11. Statistical Analysis

**Figure 1:**
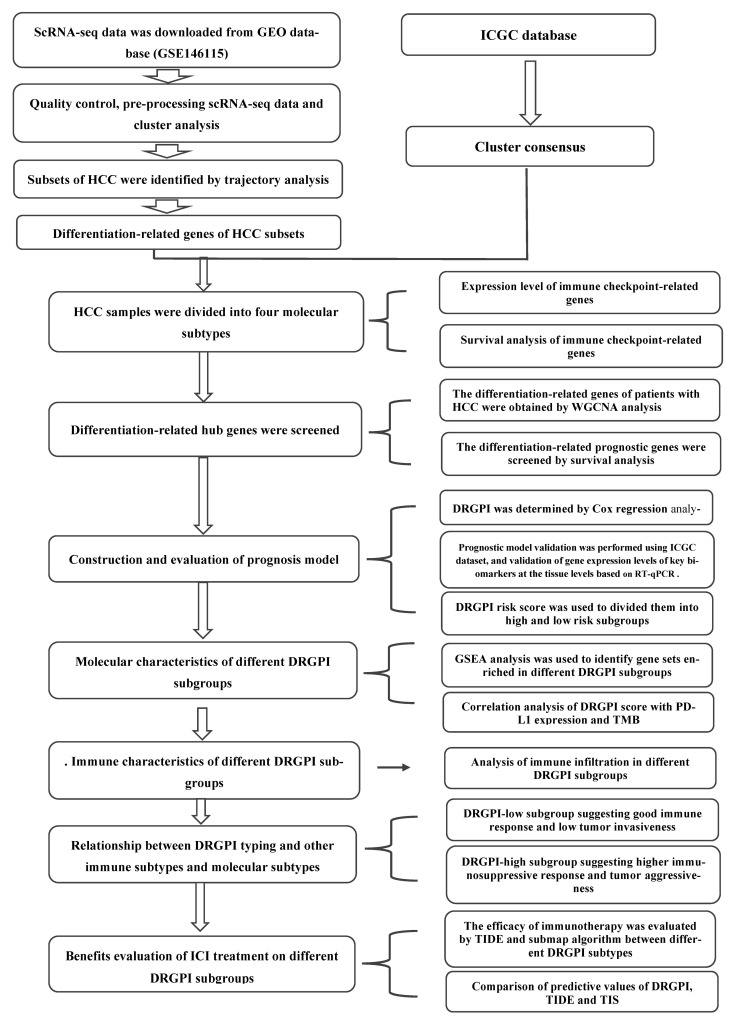
The flow sheet of this program.

All data were analyzed and plotted using R software (version 4.0.2). Log-rank test was used for plotting the survival curve, Wilcoxon rank-sum test was used for differential expression analysis, continuous variables were analyzed using the student’s *t*-test, and categorical variables were analyzed using the Chi-square test. *p* < 0.05 (two-tailed) was considered statistically significant (unless otherwise stated). The flowsheet of this program is shown in Figure 1.

## 3. Results

### 3.1. Annotation of 14 Cell Clusters Revealed High Cell Heterogeneity in HCC Preprocessing scRNA-Seq Data

**Figure 2:**
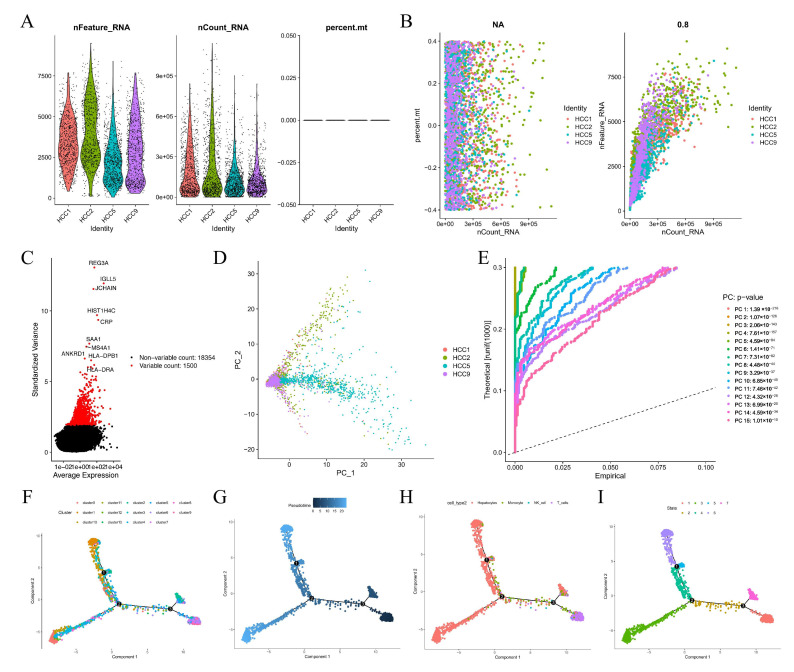
Annotation of 14 cell clusters in HCCs preprocessing scRNA-seq data displays high cell heterogeneity. (**A**) After quality control and normalization, 3200 cells from 4 HCC samples from GSE146115 were considered for further analysis. (**B**) The sequencing depth was not associated with the mitochondrial gene sequences detected, and a significant positive correlation was tested between sequencing depth and total intracellular sequences. (**C**) A total of 18354 corresponding genes and 1500 highly variable genes were displayed. (**D**) PCA analysis showed that no obvious differentiation did not occur in HCC cells. (**E**) 14 PCs were identified with an estimated *p*-value < 0.001. (**F**–**I**) Pseudo-time and trajectory analysis.

After quality control and normalization according to the matching criteria, the scRNA-seq data of 3200 cells from four HCC samples in the GSE146115 dataset were analyzed to identify differentiation-related genes (Figure 2A), and the sequencing depth was independent of the detected mitochondrial gene sequence (Figure 2B). In addition, Sequencing depth was significantly positively correlated with total intracellular sequences (R = 0.8, Figure 2B). The differential expression analysis was calculated by the “DESeq2” package of R, and the results showed that there were 18354 corresponding genes and 1500 highly variable genes (Figure 2C). Then, the top 20 strongly interaction genes in PC-1 to PC-4 were displayed as dot plots and heatmaps in Figure S1A,B.

### 3.2. Cell Trajectory Analysis Identified Seven HCC Subsets

Principal component analysis (PCA) analysis showed that HCC cells did not differ significantly (Figure 2D). Herein, the first 14 principal components (PC) with significant differences were selected for further analysis (Figure 2E). The t-distributed stochastic neighbor embedding (tSNE) analysis distinguished clear 13 clusters of 3200 HCC cells, and a total of 3883 marker genes with significantly differential expression were envaulted by differential expression analysis, and the heat map indicated the top 10% of marker genes in each cluster (Figure S1C). Thirteen clusters were assigned based on marker genes as follows: clusters 0, 1, 2, 3, 4, 8, 11, 12, and 13 were hepatocellular carcinoma cells; clusters 5 were macrophages; clusters 13 were NK-cells; and clusters 6, 7, and 9 were T-cells. Trajectory and pseudo-time analysis showed that cluster 6/7/8/9 were mainly scattered in subset I; cluster 2 was mainly scattered in subset II; cluster 5 was mainly scattered in subset III; cluster 0/4/8 were mainly scattered in subset IV; cluster 10/11/12 were mainly scattered in subset V; cluster 13 was mainly scattered in subset VI; cluster 1/3 was mainly scattered in subset VII; subsets II/IV/V/VI/VII were composed of HCC cells; subset III was composed of macrophages; subset I was composed of T-cells; subset III was composed of NK-cells (Figure 2F–I).

### 3.3. Four Molecular Subtypes in ICGC Database Based on HCC Differentiation-Related Gene (HDRG)

**Figure 3:**
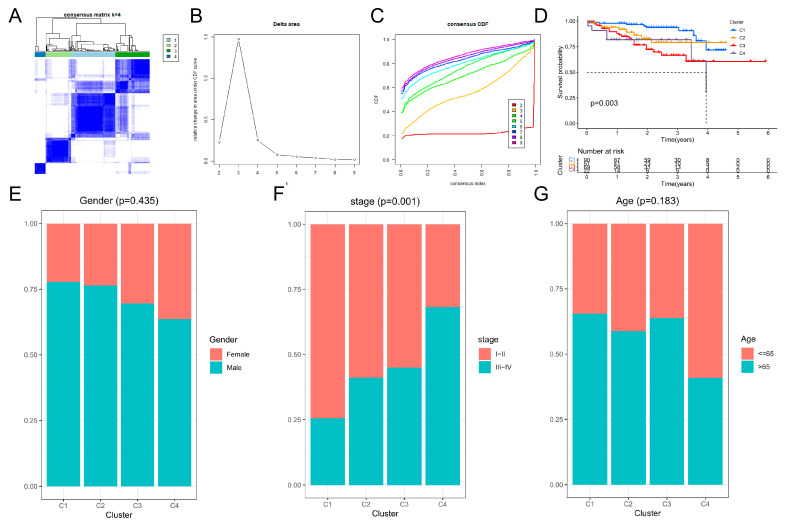
Four molecular subtypes of HCC in ICGC database based on differentiation-related genes. (**A**–**C**) Four molecular subtypes were identified at a clustering threshold of K = 9. (**D**) Kaplan–Meier analysis between the four molecular subtypes. (**E**–**G**) The proportion of clinicopathologic features among the four molecular subtypes.

Hereby, we independently applied the HDRG-based consensus clustering analysis in the ICGC cohort and identified the optimal cluster number as four molecular subtypes with a clustering threshold of maxK = 9, as shown in Figure 3A–C. Kaplan–Meier analysis revealed the significance of HDRG-based molecular subtypes in patients with HCC, and it was found that patients with subtype I (C1) had the best OS and subtype IV (C4) had the worst OS (Figure 3D, *p* = 0.003). There were no differences in gender and age distributions between subtype I (C1) and subtype IV (C4), but there were significant differences in stage I–II and stage III–IV distributions (Figure 3E–G). Furthermore, a similar expression trend was found between the up/down-regulated HDRGs in subsets I/II/III/IV and subtypes I/II/III/IV (C1/2/3/4), which manifested that subtypes I/II/III/IV (C1/2/3/4) were separately composed of subsets I/II/III/IV (Figure S2A–N).

### 3.4. Analysis of Immune Cell Infiltration and ICGs Expression Levels of Four Molecular Subtypes

CIBERSORT algorithm was performed to analyze the proportion of 22 types of immune cells in each sample in the ICGC datasets (Figure S3A), and the differential expression analysis showed that there were more T cells CD8 and M1 macrophages in the C1 subtype, more Monocytes, NK cells resting, and Mast cells resting cells in the C2 subtype, more M2 macrophages and T cells CD4 memory resting cells in the C3 subtype, and more M0 macrophages and NK cells activated in the C4 subtype (Figure S3B). Then, the immune score, stromal, and ESTIMATE scores of all subtype samples were calculated using the ESTIMATE algorithm; we found that the C1, C2, C3, and C4 subtypes’ high scores had a positive association with higher stromal score and ESTIMATE score while high score had a negative association with tumor purity (Figure S3C–F). Next, the differential expression analysis showed that 33 ICGs of four molecular subtypes had significant differences (Figure S3G), and Kaplan–Meier analysis showed that high expression of CD40LG, CD274, IL12B, PDCD1LG2, CD8A, and PTPRC was correlated with good OS (Figure S2H–M). In contrast, high expression of IL12A and YTHDF1 was associated with poor OS (Figure S3N,O).

### 3.5. Construction, Evaluation, and Validation of a DRGPI-Based Prognostic Risk Model

**Figure 4:**
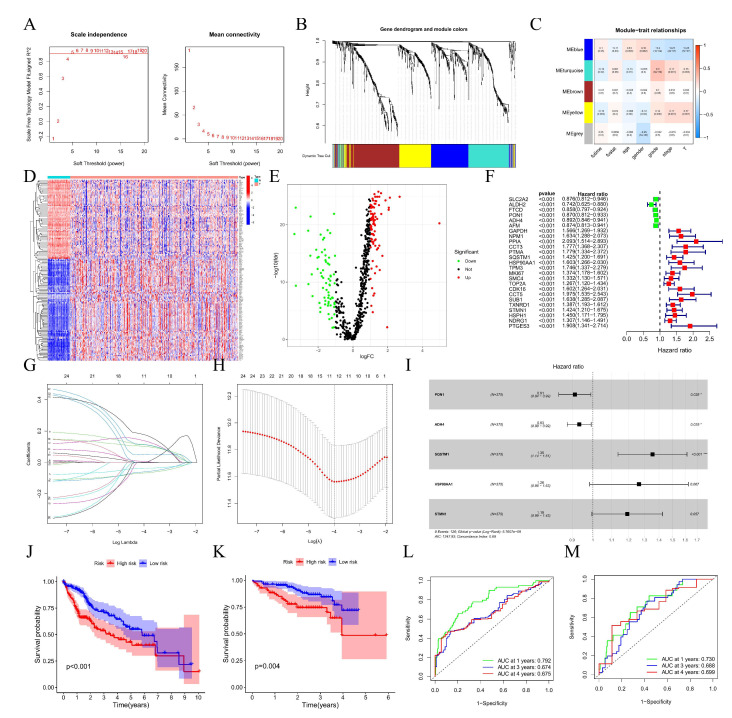
Construction, evaluation, and validation of a DRGPI-based prognostic risk model. (**A**,**B**) Based on weighted correlation network analysis, 5 modules were accessed with a soft threshold = 9. (**C**) Correlation analysis between modules and clinicopathological data of patients with HCC. (**D**) Heatmap showing the differential expression HDRGs between normal group and tumor group of HCC from TCGA. (**E**) Differential expression analysis identified 158 differentially expressed HDRGs in 3 modules, with |log2(FC)| > 1 and FDR < 0.05. (**F**) Univariate analysis of differentially expressed HDRGs. (**G**,**H**) Lasso regression analysis. (**I**) Forest plot of the multivariate Cox regression analysis establishing a differentiation-related gene prognostic index (DRGPI) in HCC. (**J**) Kaplan–Meier analysis between the low DRGPI subgroup and high DRGPI subgroup in the TCGA cohort. (**K**) Kaplan–Meier analysis between the low DRGPI subgroup and high DRGPI subgroup in the ICGC cohort. (**L**) In the TCGA cohort, the areas under the ROC curves for predicting 1-year, 3-year, and 4-year OS. (**M**) In the ICGC cohort, the areas under the ROC curves for predicting 1-year, 3-year, and 4-year OS.

**Figure 5:**
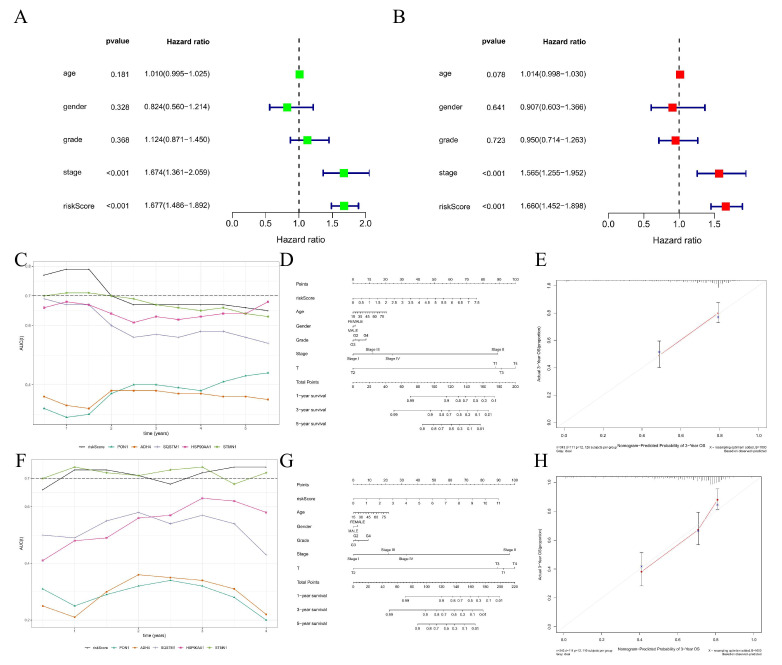
Construction and efficiency evaluation of a DRGPI-based nomogram. (**A**) Univariate Cox regression analysis. (**B**) Multivariate Cox regression analysis. (**C**) The results of AUC values of multiple indexes at multiple time points showed in TCGA cohorts. The nomogram (**D**) and its 3-year calibration curve (**E**) in TCGA cohorts. (**F**) The results of AUC values of multiple indexes at multiple time points showed in ICGC cohorts. The nomogram (**G**) and its 3-year calibration curve (**H**) in the ICGC cohort.

A total of 553 differentially expressed genes associated with HCC differentiation were obtained from overlapping them in the GEO and TCGA dataset. Meanwhile, WGCNA analysis yielded five modules, among which 158 differentially expressed HDRGs in three modules (Figure 4D,E) were significantly correlated with the pathological grade of HCC (Figure 4A,C). Kaplan–Meier analysis confirmed that the expression of 25 differentiation-related hub genes was closely correlated with the OS of patients with HCC (Figure 4F), and univariate Cox regression analysis and Lasso regression analysis were used to determine the 11 most significant differentiation-related hub genes, which were included in multivariate Cox regression analysis (Figure 5G,H). Finally, a differentiation-related gene prognostic index (DRGPI) containing five hub genes (PON1, ADH4, SQSTM1, HSP90AA1, and STMN1) was constructed by multivariate Cox regression analysis (Table 1, Figure 4I). Kaplan–Meier analysis using median DRGPI (TCGA training datasets) as the threshold suggested that patients in the low DRGPI subgroup had better OS than those in the high DRGPI subgroup (Figure 4J), and consistent results were presented in the ICGC (*n* = 232) validation datasets (Figure 4K). In addition, ROC analysis indicated that the AUC of 1-year, 3-year, and 4-year in TCGA training datasets (Figure 4L) and ICGC validation datasets (Figure 4M) were 0.792, 0.674, and 0.675 and 0.730, 0.688, and 0.699, respectively.

To further define the gene expression of DRGPI in 13 clusters, we found that PON1 was the highest in cluster 10, ADH4 was the highest in cluster 11, SQSTM1 was the highest in cluster 4, HSP90AA1 was the highest in cluster 5, and STNM1 was the highest in cluster 9 (Figure S4A,B). Correlation analysis of five genes in DRGPI showed that PON1 was positively correlated with ADH4 (r = 0.39) (Figure S4C). Meanwhile, there were significant differences in the ICGC dataset between the four subtypes in the high and low DRGPI subgroups (*p* = 0.001) (Figure S4D), and DRGPI subgroups were significantly associated with stage, grade, and T staging in TCGA cohorts (*n* = 425) with complete clinical phenotypic data (Figure S4E).

### 3.6. Validation of the Expression Levels of Five Prognostic Genes

RT-qPCR measured the expression levels of five prognostic genes in ten HCC tissues and ten adjacent normal tissues. As shown in Figure S5, the expression levels of STMN1 (Figure S5A), SQSTM1 (Figure S5B), and HSP90AA1 (Figure S5C) were elevated, while those of ADH4 (Figure S5D) and PON1 (Figure S5E) were downregulated in HCC tissues compared to the levels in the corresponding normal tissues. Here, these RT-qPCR results are consistent with the expression of HCC tissues and normal tissues in the TCGA database (Figure S5F–J).

### 3.7. Construction and Efficiency Evaluation of a DRGPI-Based Nomogram

Univariate Cox regression analysis showed that tumor staging and DRGPI were significantly correlated with the prognosis of patients with HCC (Figure 5A). Multivariate Cox regression analysis confirmed that DRGPI was an independent prognostic factor after calibrating for other clinicopathological factors (Figure 5B). The AUC values of multiple indexes at multiple time points showed that STMN1 had a better predictive effect than other single indexes in TCGA and ICGC cohorts (Figure 5C,F), respectively. Furthermore, a nomogram was constructed to predict the OS of patients with HCC at 1 year, 3 years, and 5 years by using five prognostic factors in TCGA and ICGC cohorts, respectively (Figure 5D,G). Moreover, the calibration curve indicated that the nomogram had a high accuracy in predicting the survival rate of patients with HCC (Figure 5E,H).

### 3.8. Molecular Characteristics of Different DRGPI Subgroups

**Figure 6:**
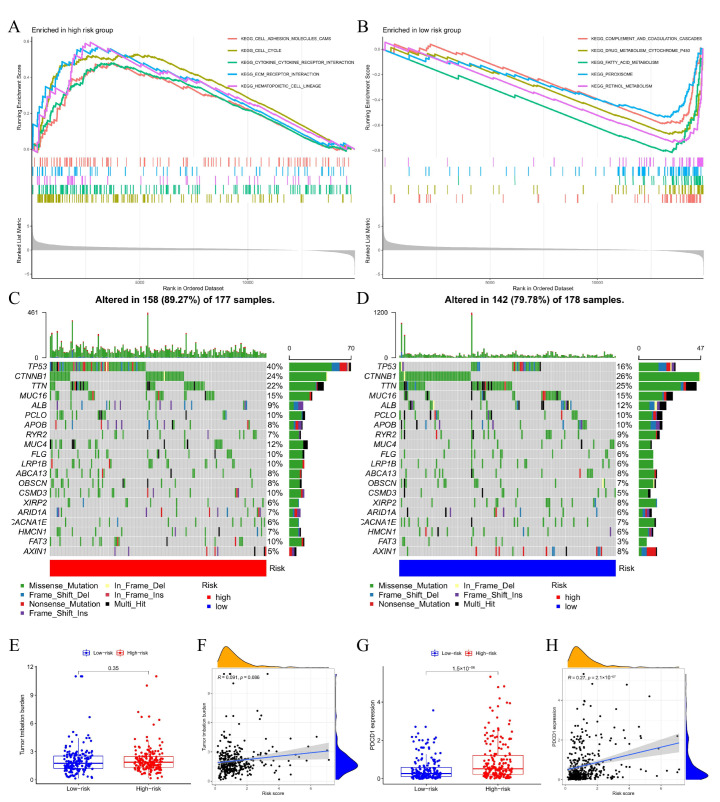
Molecular characteristics of different DRGPI subgroups. (**A**) Gene sets enriched in DRGPI-high subgroup. (**B**) Gene sets enriched in DRGPI-low subgroup. Gene mutation analysis showed that mutation count in the high DRGPI subgroup (**C**) was significantly higher than that in the low DRGPI subgroup (**D**). (**E**,**F**) Correlation analysis between DRGPI score and total mutational burden (TMB). (**G**,**H**) Correlation analysis between DRGPI score and PD-L1 expression.

GSEA analysis suggested that the samples in the high DRGPI subgroup were enriched in the pathways associated with cell proliferation and differentiation (Figure 6A). The samples in the low DRGPI subgroup were enriched in the pathways associated with immune response and inhibition of apoptosis (Figure 6B). Then, gene mutation analysis showed that the mutation count in the high DRGPI subgroup was significantly higher than that in the low DRGPI subgroup, and missense mutation was the most common mutation type, followed by meaningless deletion. Among the top four genes with the highest mutation rate in different DRGPI subgroups, TP53, CTNNB1, TTN, and MUC16 mutation rates were over 15%. The mutation of MUC4 and FAT3 was more common in the high DRGPI subgroup (Figure 6C), while the mutation of ALB was more common in the low DRGPI subgroup (Figure 6D). Next, DRGPI score was found to be significantly correlated with PD-L1 expression (r = 0.27, *p* < 0.001) (Figure 6G,H), but not with TMB (Figure 6E,F).

### 3.9. Immune Characteristics of Different DRGPI Subgroups and Their Relationship with Other Immune Subtypes

**Figure 7:**
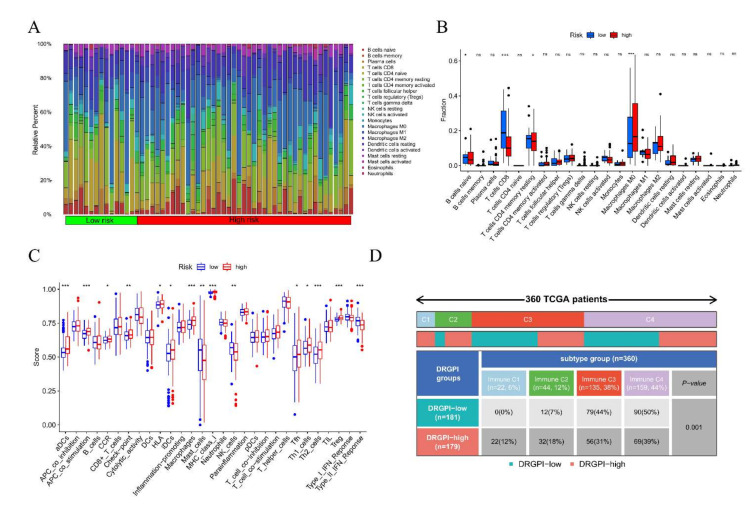
Immune characteristics of different DRGPI subgroups and their relationship with other immune subtypes. (**A**,**B**) Wilcoxon test was used to compare the distribution of immune cells in different DRGPI subgroups, and it was found that M0 macrophages were more abundant in the high DRGPI subgroup, while T cells CD4 memory resting and CD8 T cells were more abundant in the low DRGPI subgroup. (**C**) The molecular and immune-related function of different DRGPI subgroups. (**D**) The four immune subtypes had significant differences between different DRGPI subgroups (*p* = 0.001). * *p* < 0.05, ** *p* < 0.01, *** *p* < 0.001. NS, not significant.

Wilcoxon test was used to compare the distribution of immune cells in different DRGPI subgroups, and it was found that M0 macrophages were more abundant in the high DRGPI subgroup, while T cells CD4 memory resting and CD8 T cells were more abundant in the low DRGPI subgroup (Figure 7A,B). Subsequently, a few gene markers were used to define the immune and molecular functions among different DRGPI subgroups, and Mast cells, NK cells, and IFN Response were found to be more in the low DRGPI subgroup, while macrophages, tumor metastasis, and immune escape signals were found to be more in the high DRGPI subgroup (Figure 7C). Then, we described the immune landscape of HCC based on tumor and stromal compartments and summarized four immune subtypes: Wound Healing (Immune C1), Inflammatory (Immune C3), TLymphocyte Depleted (Immune C4), and IFN-gamma Dominant (Immune C2), and found that the four immune subtypes had significant differences between DRGPI subgroups (*p* = 0.001) (Figure 7D). The samples of immune subtypes C1 and C2 in the high DRGPI subgroup (12% and 18%) were higher than those in the low DRGPI subgroup (0% and 7%). Similarly, the samples of immune subtypes C3 and C4 in the low DRGPI subgroup (44% and 50%) were higher than those in the high DRGPI subgroup (31% and 39%).

### 3.10. The Benefit of ICI Therapy in Different DRGPI-Based Risk Subgroups

**Figure 8:**
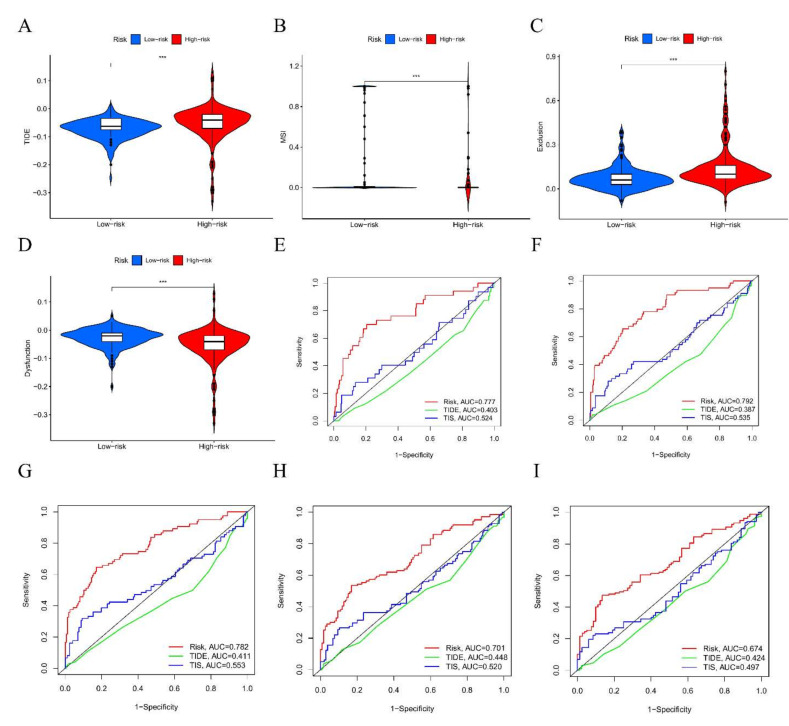
The benefit of ICI therapy in different DRGPI-based risk subgroups. TIDE (**A**), MSI (**B**), T cell exclusion (**C**), and dysfunction score (**D**) in different DRGPI subgroups. The scores between the two DRGPI subgroups were significantly different from TCGA (*** *p* < 0.001). (**E**–**I**) ROC analysis was used to verify the prognostic value of DRGPI in the immunotherapy response of ICI; the results indicated that the AUC index of DRGPI was higher than that of TIDE and TIS at 6/12/18/24/36 months of follow-up.

Our study found that TIDE scores in the high DRGPI subgroup were higher than those in the low DRGPI subgroup, suggesting that patients in the low DRGPI subgroup could benefit more from ICI therapy and have a better prognosis than those in the high DRGPI subgroup (Figure 8A). It was also found that the low DRGPI subgroup had higher microsatellite instability (MSI) scores (Figure 8B), while the high DRGPI subgroup had a higher T cell exclusion score (Figure 8C), and the low DRGPI subgroup had higher T cell dysfunction (Figure 8D). ROC analysis was used to verify the prognostic value of DRGPI in the immunotherapy response of ICI, and the results indicated that the AUC index of DRGPI was higher than that of TIDE and TIS at 6/12/18/24/36 months of follow-up (Figure 8E–I). Therefore, we believed that the predictive value of DRGPI was greater than that of TIS and TIDE in the TCGA cohort.

## 4. Discussion

In the last few years, with the understanding of the tumor immune microenvironment, immunotherapy represented by ICIs or neoadjuvant therapy has shown good therapeutic outcomes in colon cancer [22], melanoma, and other cancers [23], as well as promising prospects in the treatment of HCC. Nevertheless, the heterogeneity of the tumor immune microenvironment and many other factors limit the therapeutic effects of HCC, and the efficacy is far from reaching our expectations [24]. Emerging evidence also suggests that the heterogeneity of the immune microenvironment is related to pathological grading, clinical staging, primary or metastatic, and immunotherapy response and significantly impacts the prognosis of patients with HCC [25,26].

Just a few years ago, the advent of scRNA-seq technology opened a new avenue to explore the cell types and cell differentiation status in tumors, as well as to analyze differentially expressed genes, especially in refractory tumors [27,28]. However, there are no studies based on scRNA-seq to predict the therapeutic effect of ICI for HCC to provide a multi-directional exploration perspective for the therapeutic targets of HCC, which also highlights the necessity of identifying prognostic biomarkers in HCC [29]. Hereby, we explored the genetic heterogeneity in the tumor microenvironment of HCC from the perspective of cell differentiation trajectory to confirm which patients with HCC can benefit from the treatment of ICI and provide a reference for individualized therapy. Ultimately, a DRGPI constructed by our team proved to be an effective biomarker for predicting immunotherapy response and survival time in patients with HCC, and this DRGPI may also be a key hub in the progression of HCC.

Herein, based on the data from the scRNA-seq of HCC, differentiation-related genes that affect the multi-gene heterogeneity in the immune microenvironment of HCC were explored through cell differentiation trajectory. Further, 25 key differentiation-related genes affecting the OS of patients with HCC were identified by WGCNA, and a DRGPI and nomogram containing five genes (PON1, ADH4, SQSTM1, HSP90AA1, and STMN1) were constructed by Cox regression analysis. Furthermore, ROC analysis showed that the AUC value of DRGPI was greater than 0.7 in predicting 3-year survival of patients with HCC in both TCGA and ICGC cohorts, and patients with HCC at low DRGPI risk had better survival than those at high DRGPI risk, suggesting that DRGPI is an effective prognostic biomarker.

Studies have shown that PON1, which is mainly synthesized by the liver [30], has a protective effect on oxidative stress against oxidative phosphatase [31], is associated with the severity of liver damage, and plays a vital role in the development of chronic liver disease into cirrhosis or HCC [32]. Other studies have suggested that ADH4, as a member of the ADH family, may also be a potential protective biomarker for monitoring the prognosis of HCC [33,34]. SQSTM1, also known as P62 protein, is one of the autophagy-related proteins [35], which is involved in various signal transduction processes in vivo and is degraded during autophagy and apoptosis [36], and the accumulation of SQSTM1 inhibits autophagy and promotes tumor progression [37]. The study of X. Xiang, W. Shi, Y. Fu et al. found that heat shock protein HSP90AA1 was highly expressed in the plasma of patients with HCC, which promoted the cell cycle progression and migration of HCC cells, and could serve as a potential biomarker for the progression of HCC induced by hepatitis C virus [38,39,40]. In addition, Liu et al. showed that a nomogram constructed from the mRNA expression of plasma HSP90AA1 could predict the risk of breast cancer incidence and metastasis [41]. Additionally, tubulin STMN1 is up-regulated and has de-stabilizing activity in various tumor tissues [42], which is associated with the proliferation, invasion, and migration of gastric cancer [43], ovarian cancer [44], and HCC [45]. Interestingly, in the DRGPI risk assessment model constructed by our team, the coefficients of PON1 and ADH4 were negative. In contrast, the coefficients of SQSTM1, HSP90AA1, and STMN1 were positive, which was consistent with the reported function of promoting or suppressing cancer, indicating that DRGPI is a valuable prognostic biomarker for HCC patients.

To explore the mutation characteristics of the DRGPI subgroup, we analyzed that the largest mutation difference between the two groups was TP53 mutation, whose mutation rate in DRGPI high-risk group was higher than that in DRGPI low-risk group (40% vs. 16%). TP53 mutations are known to be the most common genetic mutations in cancer, and patients with a high rate of P53 mutations have stronger tumor aggressiveness and poorer prognosis, especially in patients with HCC, which is consistent with our findings. Additionally, the CIBERSORT algorithm was applied to investigate the infiltration of immune cells in the DRGPI subgroup, and it was found that CD8 T cells were more enriched in the low DRGPI subgroup, while M0 macrophages were more enriched in the high DRGPI subgroup. Numerous studies have shown that intensive infiltration of CD8 T cells in the tumor immune microenvironment predicts a good prognosis [46,47]. Macrophages, as the primary immune-infiltrating cells in tumor tissues, are key cell types in most tumors’ regulation of tumor and inflammation [48,49]. Meanwhile, other studies have also shown that M0 macrophage enrichment is conducive to tumor growth and the development of aggressive phenotypes [50,51] and is associated with poor prognosis of glioblastoma [52], HCC [53], breast tumor [54], etc., and our research supports this conclusion as well. Furthermore, we found that samples from the low DRGPI subgroup were more likely to activate the complement pathway to regulate immune and physiological functions and participate in more cell adhesion molecule pathways to affect cell activation, signal transduction, and cell proliferation and differentiation, suggesting that the subgroup with high DRGPI has the characteristics of inhibiting immunotherapy response and promoting tumor progression in HCC patients treated with hepatectomy.

Studies have confirmed that the TIDE score was performed to evaluate the potential clinical efficacy of ICI therapy in different DRGPI subgroups. The higher TIDE prediction score indicates that patients may benefit less from ICI therapy and have a shorter survival time [55]. Similarly, we found that DRGPI can also be used to predict ICI therapy response and differentiate the heterogeneity of the immune microenvironment in HCC. Here, to evaluate the efficacy of DRGPI score in the therapy of ICI in the individual patient with HCC, AUC value at different follow-up time points was used to evaluate the efficacy of DRGPI, TIDE, and TIS in predicting the immunotherapy response of ICI in the TCGA dataset. Excitingly, DRGPI performed better than the other two indicators at predicting immune response at different follow-up time points. More importantly, DRGPI was made up of just five genes, which was easier than testing TIDE and TIS. Additionally, compared with the high DRGPI subgroup, patients in the low DRGPI subgroup had lower microsatellite instability (MSI) score, lower T cell exclusion score, and higher T cell dysfunction, suggesting that patients in the low DRGPI subgroup had lower immune escape levels. It has been confirmed that MSI is prevalent in HCC, and the MSI of patients with HCC may be immunogenic neoantigens, which can induce massive infiltration of cytotoxic T cells, and their immunogenic neoantigens are sensitive to anti-PD-1 therapy in patients with HCC [56,57], and our research reaffirms this opinion.

Further, the prognostic model and nomogram established according to DRGPI have been well validated in TCGA and ICGC cohorts. Meanwhile, we found that the survival time of the low DRGPI subgroup was significantly higher than that of the high DRGPI subgroup, and logistic regression analysis showed that the DRGPI risk score could be used as an independent prognostic factor in an integrated model that incorporates clinically relevant features, and ROC analysis revealed the strong prognostic value of DRGPI in patients with HCC. Unfortunately, our study is also accompanied by some shortcomings: (i) the TCGA database is mainly derived from U.S. or European populations, and it is unclear whether this applies to Asian populations; (ii) although the efficacy of DRGPI has been validated in the ICGC database, the lack of basic experimental studies has prevented the details of its molecular mechanism from being elucidated.

## 5. Conclusions

In conclusion, DRGPI is a promising prognostic biomarker related to the HCC cell differentiation trajectory. DRGPI subtype typing may help identify the immune microenvironment and molecular characteristics of HCC patients and predict whether ICI therapy will be beneficial. DRGPI may be a potential prognostic indicator for ICI therapy; if it can be clarified in future clinical and molecular mechanistic experiments, it will bring great clinical application prospects.

## Figures and Tables

**Table 1:** Multivariate Cox proportional hazards regression model including the key members of the DRGPI for overall survival in patients with HCC.

Gene	Coefficient	HR	95%CL		*p*-Value
			Lower	Upper	
PON1	−0.103	0.90	0.83	0.98	0.014
ADH4	−0.067	0.94	0.88	0.99	0.033
SQSTM1	0.307	1.36	1.14	1.62	0.001
HSP90AA1	0.252	1.29	1.00	1.65	0.048
STMN1	0.180	1.20	1.00	1.43	0.049

HCC—hepatocellular carcinoma; DRGPI—differentiation-related gene prognostic index; HR—hazard ratio; CI—confidence interval.

## Data Availability

Publicly available datasets were analyzed in this study. The datasets can be found in The Cancer Genome Atlas (TCGA) at https://portal.gdc.cancer.gov (accessed on 18 July 2021), ICGC (https://dcc.icgc.org/donor.LIRI-JP.tsv.gz) (accessed on 20 July 2021) dataset, and GEO database (GSE146115) (https://www.ncbi.nlm.nih.gov */*geo*/*) (accessed on 15 July 2021).

## Supplementary Materials

The following supporting information can be downloaded at: https://www.mdpi.com/article/10.3390/cells11152302/s1, Figure S1: Dot plots and heatmaps Amino acid showing the top 20 strongly interaction genes in PC-1 to PC-4; Figure S2: The same expression trend was found between the up/down-regulated DRGs in subsets I/II/III/IV and the subtypes I/II/III/IV; Figure S3: Analysis of immune cell infiltration and ICGs expression levels of four molecular subtypes; Figure S4: Correlation analysis and difference analysis of key genes in DRGPI were performed; Figure S5: Expression levels of 5 differentiation-related genes of prognostic signature in HCC tissues and corresponding normal tissues by RT-PCR; Table S1: Sequences of gene-specific primers used for real-time RT-PCR.

## Abbreviations

HCC: hepatocellular carcinoma; DRGPI, differentiation-related gene prognostic index; HR, hazard ratio; CI, confidence interval; ICI, immune checkpoint inhibitor; TCGA, The Cancer Genome Atlas; ICGC, International Cancer Genome Consortium; GEO, Group on Earth Observations; ROC, receiver operating characteristic; TCGA, The Cancer Genome Atlas; TIDE, Tumor Immune Dysfunction and Exclusion; AUC, area under curve; CIBERSORT, cell type identification by estimating relative subsets of RNA transcripts; HDRG, HCC differentiation-related gene; ORR, objective remission rate.
