# Supplementary material for: A Differentiation-Related Gene Prognostic Index Contributes to Prognosis and Immunotherapy Evaluation in Patients with Hepatocellular Carcinoma
Source: Cells. 2022 Jul 26;11(15):2302. doi: 10.3390/cells11152302 (PMC9367442; doi:10.3390/cells11152302)

Supplementary Table S1. Sequences of gene-specific primers used for real-time RT-PCR.

Gene	Primer Sequence
PON1 forward	5′-AGGAGACCTTTGGGTTGG-3′
PON1 Reverse	5′-ACTGTGCCATTTTCTGCAT -3′
ADH4 forward	5'-TGGATTTTGCCCTTGACT-3 '
ADH4 Reverse	5'-GGCCGATTATTAGCTCCTC-3'
SQSTM1 forward	5'-CTGCCTTGTTTCACCTTCC-3'
SQSTM1 Reverse	5'-GCCCTGGCATTGTTCTTAC-3'
HSP90AA1 forward	5'-GGTTACATGGCAGCAAAGA-3'
HSP90AA1 Reverse	5'-AGCGCAGTTTCATAAAGCA-3'
STMN1 forward	5'-ATCCCAGTTGATTGTGCAG-3'
STMN1 Reverse	5'-CGCTTCTCCAGTTCTTTCA-3'

Figure S1. Dot plots and heatmaps Amino acid showing the top 20 strongly interaction genes in PC-1 to PC-4.
(A) The dot plots show the top 20 significantly correlated genes in each component. **(B)** The heatmaps show the expression patterns of the top 20 significantly correlated genes in each component. **(C)** The heat map indicated the top 10% of marker genes in each cluster.

Figure S2.
(A-N) The same expression trend was found between the up/down-regulated DRGs in subsets I/II/III/IV and the subtypes I/II/III/IV (C1/2/3/4).

Figure S3. Analysis of immune cell infiltration and ICGs expression levels of four molecular subtypes.
(A, B) CIBERSORT algorithm was performed to analyze the proportion of 22 types of immune cells in each sample in the ICGC datasets. The immune score **(C)**, stromal score **(D)**, ESTIMATE **(E)** and tumor purity scores **(F)** of all each subtype samples were calculated using the ESTIMATE algorithm between C1, C2, C3 and C4 subtypes. **(G)** The differential expression analysis showed that 33 ICGs of four molecular subtypes had significant differences. Kaplan-Meier analysis showed that high expression of CD40LG **(H)**, CD274 **(I)**, IL12B **(J)**, PDCD1LG2 **(K)**, CD8A **(L)**, PTPRC **(M)** was correlated with good OS, while high expression of IL12A **(N)** and YTHDF1 **(O)** was associated with poor OS.

Figure S4. Correlation analysis and difference analysis of key genes in DRGPI were performed. (A, B) Expression levels of DRGPIs in 13 clusters. **(C)** Correlation analysis of 5 genes in DRGPI showed that PON1 was positively correlated with ADH4 (r=0.39). **(D)** In the ICGC dataset, there were significant differences between the 4 subtypes in the high and low DRGPI subgroups (P =0.001). **(E)** DRGPI subgroups was significantly associated with stage, grade, and T staging in TCGA cohorts with complete clinical phenotypic data.

Figure S5. Expression levels of 5 differentiation-related genes of prognostic signature in HCC tissues and corresponding normal tissues by RT-PCR. The expression levels of STMN1 **(A)**, SQSTM1 **(B)**, and HSP90AA1 **(C)** were elevated, while those of ADH4 **(D)** and PON1 **(E)** were downregulated in HCC tissues compared to the levels in the corresponding normal tissues. Here, these RT-qPCR results are consistent with the expression of HCC tissues and normal tissues in the TCGA database **(F-J)**.

Figure S1

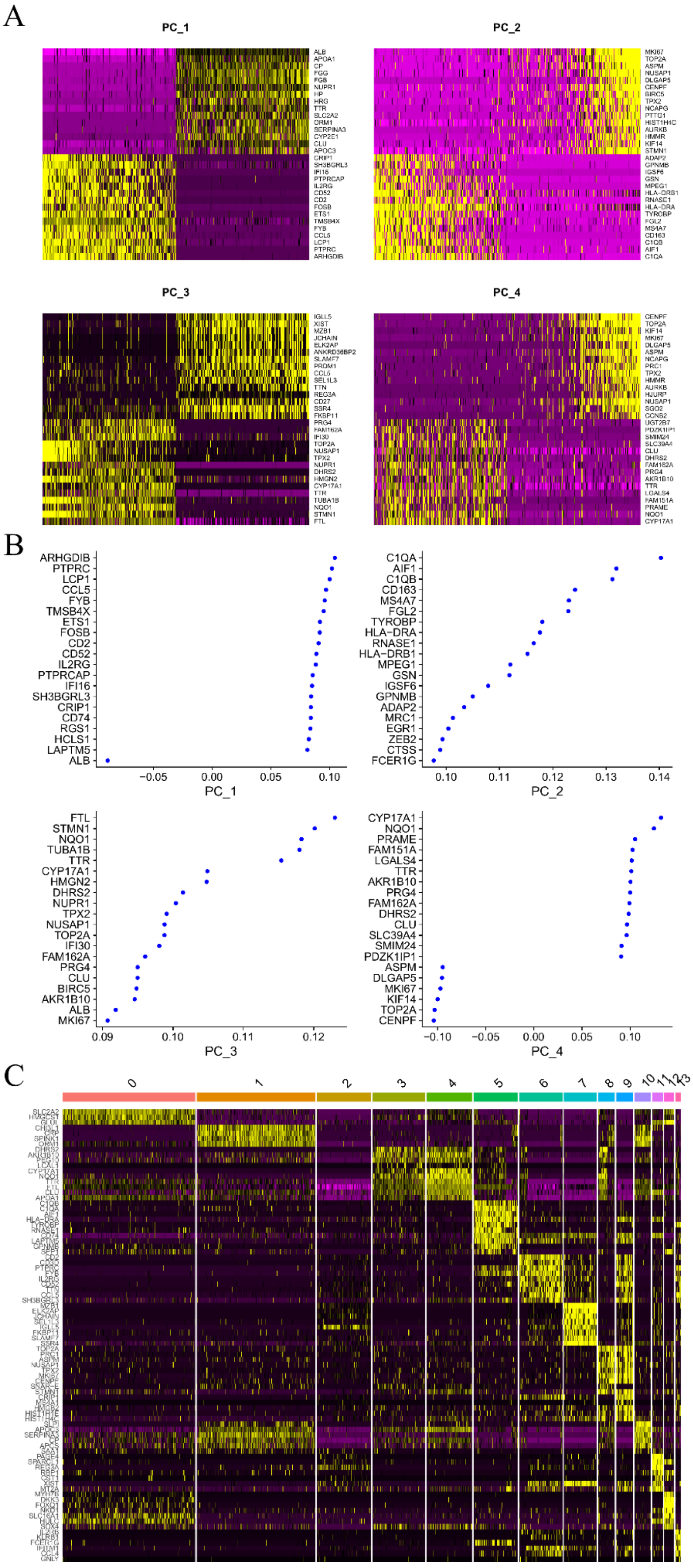
A
PC_1
PC_2
PC_3
PC_4
B
ARHGDIB
PTPRC
LCP1
CCL5
FYB
TMSB4X
ETS1
FOSB
CD2
CD52
IL2RG
PTPRCAP
IFI16
SH3BGRL3
CRIP1
CD74
RGS1
HCLS1
LAPTM5
ALB
-0.05 0.00 0.05 0.10
PC_1
C1QA
AIF1
C1QB
CD163
MS4A7
FGL2
TYROBP
HLA-DRA
RNASE1
HLA-DRB1
MPEG1
GSN
IGSF6
GPNMB
ADAP2
MRC1
EGR1
ZEB2
CTSS
FCER1G
0.10 0.11 0.12 0.13 0.14
PC_2
FTL
STMN1
NQO1
TUBA1B
TTR
CYP17A1
HMGN2
DHRS2
NUPR1
TPX2
NUSAP1
TOP2A
IFI30
FAM162A
PRG4
CLU
BIRC5
AKR1B10
ALB
MKI67
0.09 0.10 0.11 0.12
PC_3
CYP17A1
NQO1
PRAME
FAM151A
LGALS4
TTR
AKR1B10
PRG4
FAM162A
DHRS2
CLU
SLC39A4
SMIM24
PDZK1IP1
ASPM
DLGAP5
MKI67
KIF14
TOP2A
CENPF
-0.10 -0.05 0.00 0.05 0.10
PC_4
C
0 1 2 3 4 5 6 7 8 9 10 11 12 13

Figure S2

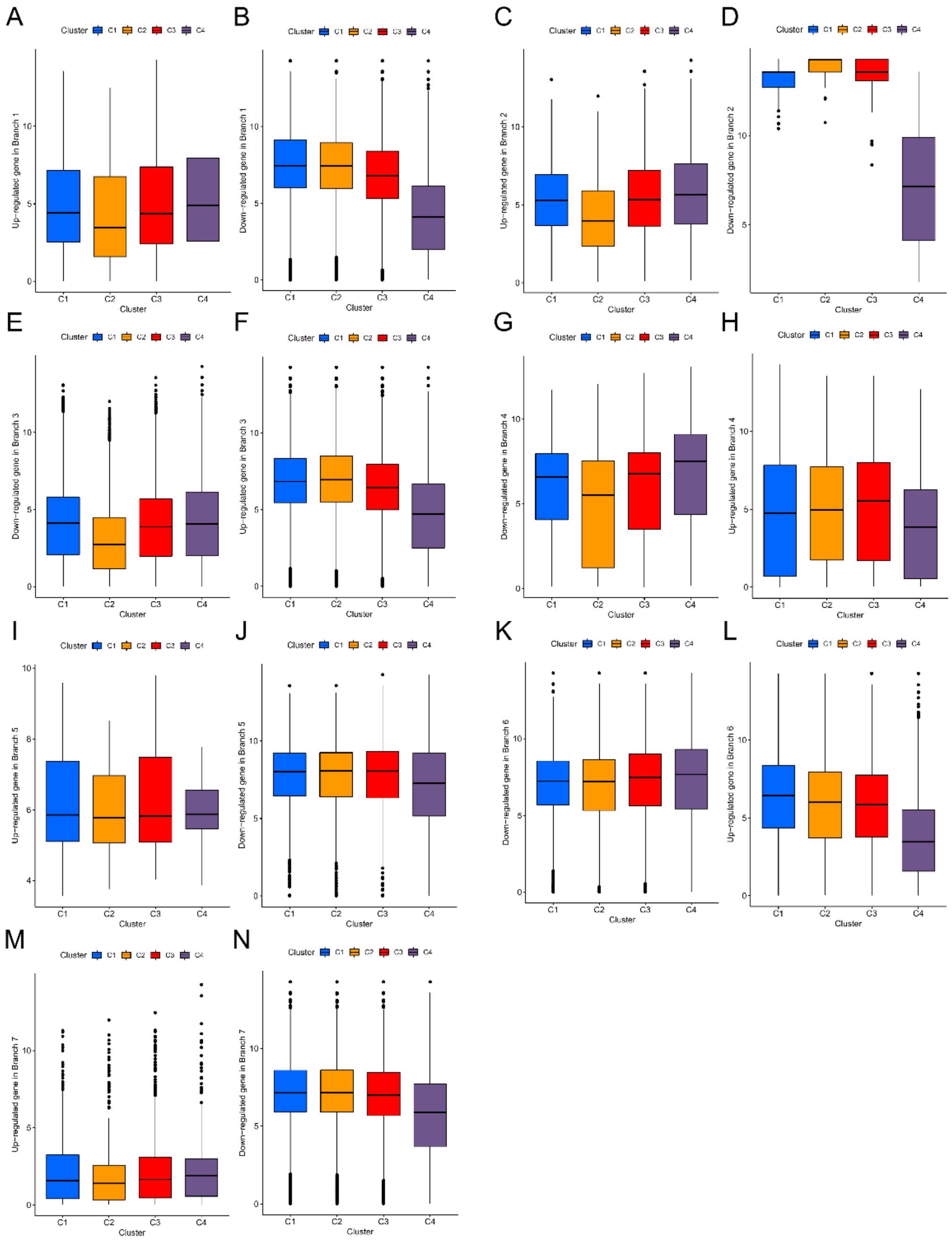
A
B
C
D
E
F
G
H
I
J
K
L
M
N
Cluster C1 C2 C3 C4
Up-regulated gene in Branch 1
Down-regulated gene in Branch 1
Up-regulated gene in Branch 2
Down-regulated gene in Branch 2
Down-regulated gene in Branch 3
Up-regulated gene in Branch 3
Down-regulated gene in Branch 4
Up-regulated gene in Branch 4
Up-regulated gene in Branch 5
Down-regulated gene in Branch 5
Down-regulated gene in Branch 6
Up-regulated gene in Branch 6
Up-regulated gene in Branch 7
Down-regulated gene in Branch 7
Cluster

Figure S3

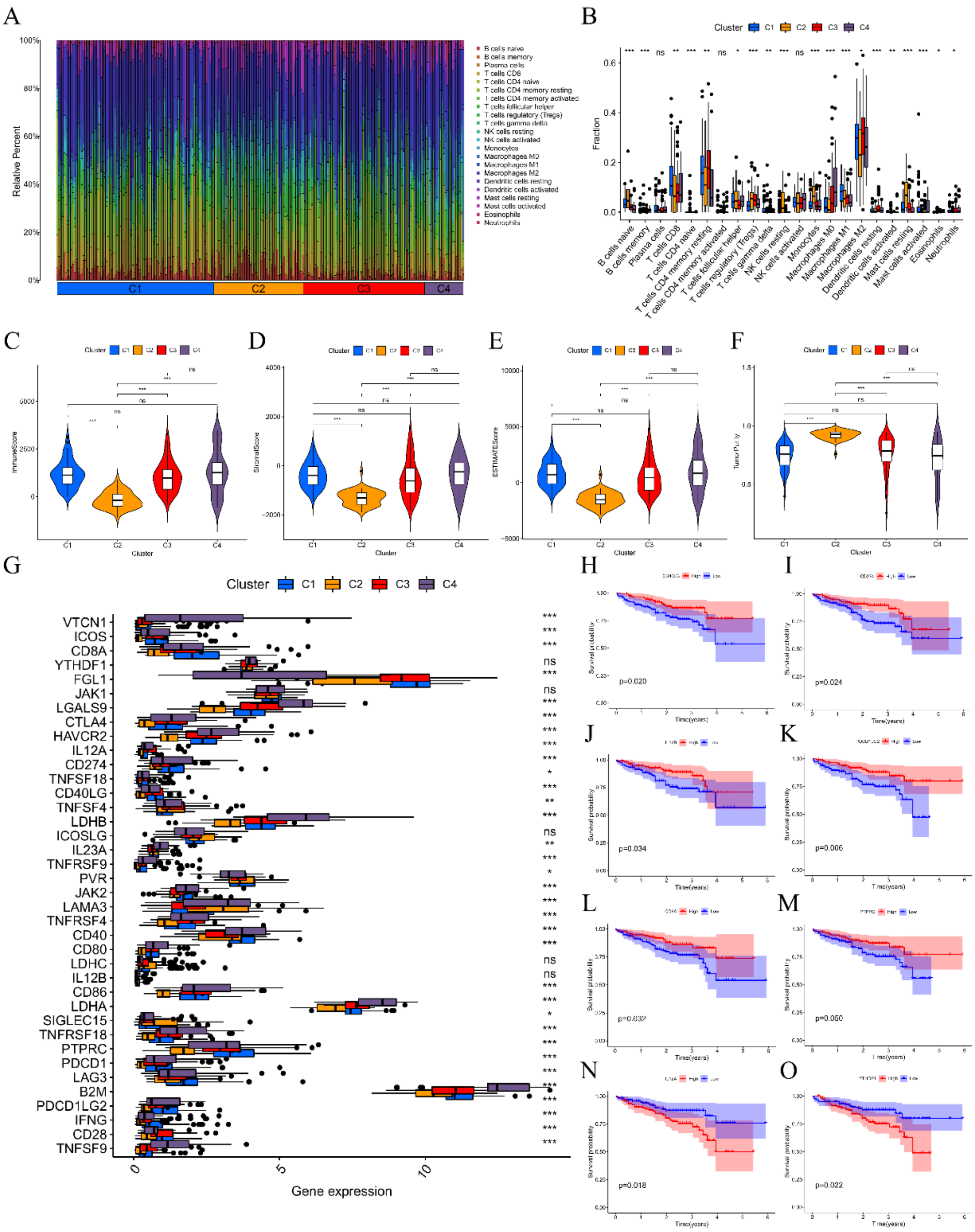

A
B
C
D
E
F
G
H
I
J
K
L
M
N
O
Relative Percent
C1
C2
C3
C4
Cluster
Fraction
Gene expression
VTCN1
ICOS
CD8A
YTHDF1
FGL1
JAK1
LGALS9
CTLA4
HAVCR2
IL12A
CD274
TNFSF18
CD40LG
TNFSF4
LDHB
ICOSLG
IL23A
TNFRSF9
PVR
JAK2
LAMA3
TNFRSF4
CD40
CD80
LDHC
IL12B
CD86
LDHA
SIGLEC15
TNFRSF18
PTPRC
PDCD1
LAG3
B2M
PDCD1LG2
IFNG
CD28
TNFSF9
p=0.020
p=0.024
p=0.034
p=0.006
p=0.032
p=0.050
p=0.018
p=0.022

Supplementary Fig. S4

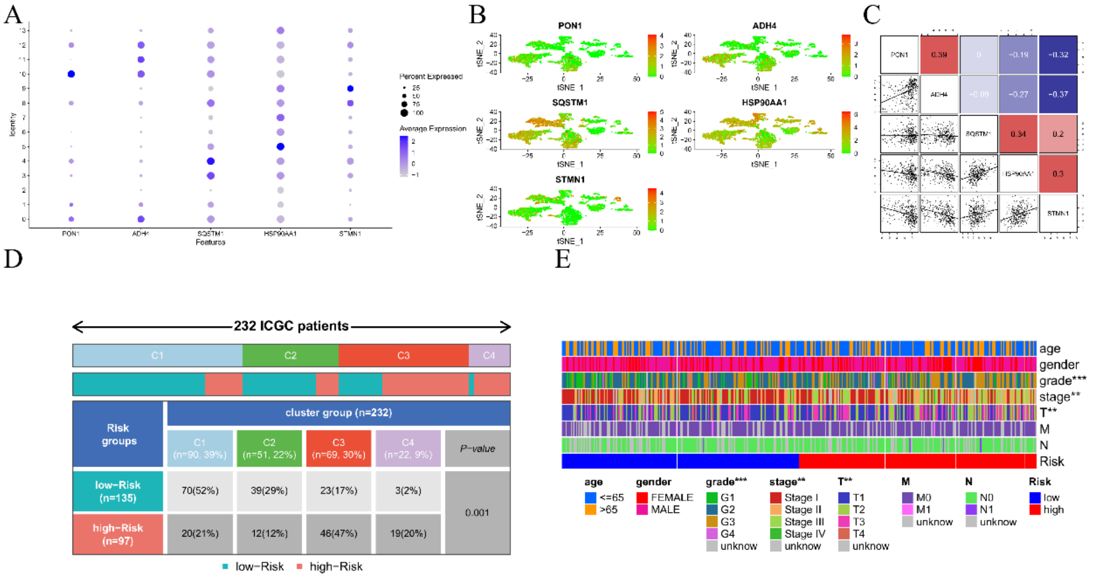

Supplementary Fig. S5

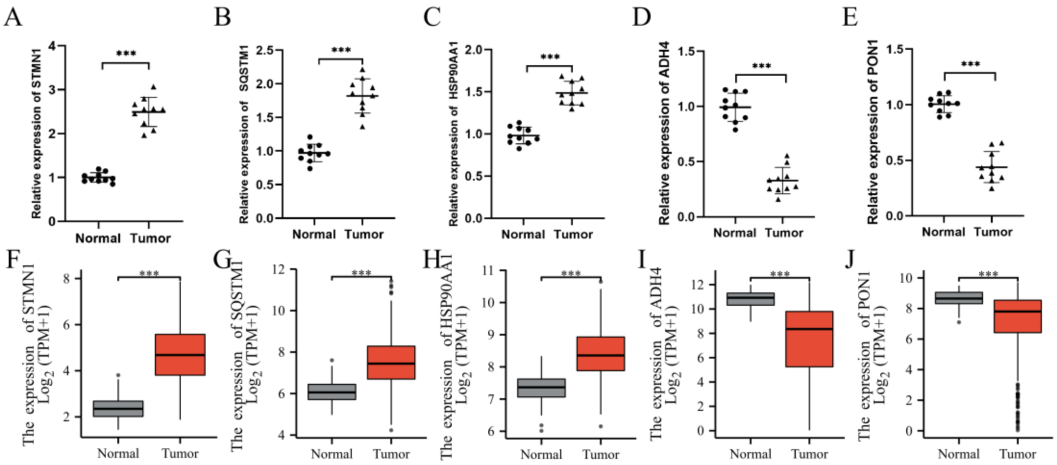

Supplement: Supplementary file 1 [file cells-11-02302-s001.zip › cells-1787688-supplementary.pdf]
